# *Lupinus mutabilis* Extract Exerts an Anti-Diabetic Effect by Improving Insulin Release in Type 2 Diabetic Goto-Kakizaki Rats

**DOI:** 10.3390/nu10070933

**Published:** 2018-07-20

**Authors:** Silvia Zambrana, Lena C. E. Lundqvist, Orlando Mamani, Sergiu-Bogdan Catrina, Eduardo Gonzales, Claes-Göran Östenson

**Affiliations:** 1 Instituto de Investigaciones Farmaco Bioquimicas, Universidad Mayor de San Andres, Avenida Saavedra, La Paz 2224, Bolivia; you_dinhi@hotmail.com (O.M.); eduardo.gonzales@gmail.com (E.G.); 2 Department of Molecular Medicine and Surgery, Karolinska Institutet, Karolinska University Hospital, Solna (L1:00), SE-171 76 Stockholm, Sweden; Sergiu-Bogdan.Catrina@ki.se (S.-B.C.); Claes-Goran.Ostenson@ki.se (C.-G.Ö.); 3 Department of Molecular Sciences, Swedish University of Agricultural Sciences, P.O. Box 7015, SE-750 07 Uppsala, Sweden; lena.lundqvist@slu.se; 4 Department of Endocrinology and Metabolism, Karolinska University Hospital, 141 86 Stockholm, Sweden; 5 Centrum for Diabetes, Academic Specialist Centrum, 14186 Stockholm, Sweden

**Keywords:** *Lupinus mutabilis*, nutraceutical, natural product, diabetes mellitus type 2 diabetes, insulin secretion, Goto-Kakizaki rats

## Abstract

*Lupinus mutabilis* (*LM*) is a legume part of Bolivian traditional diet that has a nutraceutical property reducing blood glucose levels. The prevalence of type 2 diabetes is increasing worldwide thus; the search for novel anti-diabetic drugs is needed. Based on its traditional use, we evaluated the anti-diabetic effect of *LM* in the spontaneously diabetic Goto-Kakizaki (GK) rat, a model of type 2 diabetes and in Wistar (W) rats as healthy control. *LM* seeds hydroethanolic extract, analyzed by gas chromatography-mass spectrometry and high-performance liquid chromatography-high resolution mass spectrometry, is a complex mixture of volatile and non-volatile components. A single oral administration of *LM* extract (2000 mg/kg b.w.) improved glucose tolerance during the oral glucose tolerance test (OGTT) (30–120 min) in GK and W rats (*p* < 0.0001). The long-term treatment with *LM* (1000 mg/kg b.w.), for 21 days, improved the area under the curve (AUC) of glucose during OGTT at day 20, in both GK (*p* < 0.01) and W rats (*p* < 0.01). The HbA1c (GK rats, *p* < 0.05 and W rats, *p* < 0.0001) and the non-fasting glucose (GK rats, *p* < 0.05) were also reduced. *LM* increased both serum insulin levels (2.4-fold in GK rats and 2.5-fold W rats), and the glucose-induced (16.7 mM glucose) insulin release in isolated islets from treated animals (6.7-fold in GK rats, and 6.6-fold in W rats). Moreover, *LM* (10 mg/mL) stimulated *in vitro* glucose induced (16.7 mM glucose) insulin release in batch incubated GK and W rat islets (*p* < 0.0001). In perifused GK rat islets, insulin release in 16.7 mM glucose was increased 95.3-fold compared to untreated islets (*p* < 0.0001), while no significant differences were found in perifused W rat islets. The *LM* mechanism of action, evaluated using inhibitory compounds of the insulin secretion pathway, showed that *LM*-dependent insulin secretion was reduced 42% by diazoxide (*p* < 0.001), 70% by nifedipine (*p* < 0.001), 86.7% by H89 (*p* < 0.0001), 70.8% by calphostine-C (*p* < 0.0001) and 93% by pertussis toxin (*p* < 0.0001). A similar effect was observed in W rats islets. Our findings provide evidence that *LM* has an anti-diabetic effect through stimulation of insulin release. The effect is-dependent on L-type calcium channel, protein kinase A and C systems, and G protein-coupled exocytosis and is partially mediated by K-ATP channels.

## 1. Introduction

Diabetes Mellitus Type 2 (T2DM) is a health problem that has been increased worldwide and its prevalence, according to the International Diabetes Federation (IDF), is estimated to reach 642 million people by 2040 [1]. T2DM is a metabolic disorder with chronic hyperglycemia due to impaired insulin secretion and decreased insulin sensitivity of multiple etiologies [2,3,4]. The chronic hyperglycemia can lead to the development of diabetes complications i.e., nephropathy, retinopathy, neuropathy, and macrovascular damage [5,6]. Thus, blood glucose control is the main target of new anti-diabetic drug studies. 

Natural sources have offered anti-diabetic non-toxic alternatives with less or lack of side effects [5,7,8,9]. Recently, an increasing interest has been focused on nutraceuticals, that are food plants with a pharmaceutical benefit beyond their nutritional value [10,11,12,13]. Thus, based on the Bolivian traditional use, we selected the *Lupinus mutabilis* (*LM*) seeds to evaluate its anti-diabetic effect. *LM*, common name tarwi, is a legume usually consumed as cooked seeds [14,15], and is traditionally used to reduce glycemia after the meal [15]. Clinical trials showed that *LM* reduces glycemia in slightly hyperglycemic subjects [16], and cooked *LM* seeds reduce glycemia in patients with type 2 diabetes, an effect related to its alkaloid content [17]. 

The Goto-Kakizaki rat (GK) is a non-obese model of T2DM, originated from Wistar (W) rat by repeated inbreeding of animals with impaired glucose tolerance mainly due to impaired β-cell function on a background of polygenic inheritance. Similar β-cell defects have also been shown in islets isolated from T2DM patients and the polygenic nature of diabetes heredity in the GK rat may resemble the genetic basis of T2DM in humans [18,19,20,21].

The aim of this study was to characterize the anti-diabetic effect of *LM* in Goto-Kakizaki rats (GK) that is a type 2 diabetic model. Wistar rats (W) were used as control, healthy rats. The mechanism of insulin release was evaluated in pancreatic islets isolated from both GK and W rats. 

## 2. Materials and Methods 

### 2.1. Animals

Male rats (150–200 g) were used. GK rats, originated from glucose intolerant W rats, were bred in the animal facility of the Molecular Medicine and Surgery department of the Karolinska Institutet [18], and healthy W rats (150–200 g) purchased from a commercial breeder (Charles River, Sweden) were used as non-diabetic control animals. Animals were kept at 22 °C with alternating 12 h light-dark cycle and had free access to food and fresh water. Experiments were done after one week of adaptation in the experimentation unit. The study was approved by the Laboratory Animal Ethics Committee of the Karolinska Institutet (approval Dnr. N50/2014). 

### 2.2. Plant Material

Plant specimen was collected from local producers from Ancoraimes municipality, Omasuyos Province, La Paz, Bolivia (latitude 15°55′19.3″ S and longitude 68°53′50.1″ W). One voucher specimen (No. EG-1, Fabaceae) was identified and certified by the Herbario Nacional de Bolivia from Universidad Mayor de San Andres (UMSA) and has been deposited in the Area de Farmacologia of the Instituto de Investigaciones Farmaco Bioquimicas, UMSA, La Paz, Bolivia.

### 2.3. Plant Extract Preparation

The hydroethanolic extract of *LM* was prepared with 200 g of *LM* seeds macerated with 250 mL of 70% ethanol for 48 h. To maximize the yield, the maceration procedure was repeated five times. Ethanol solvent was evaporated using a rotary evaporator (Heidolph, Schwabach, Germany) and the water fraction was dried under pressure in a freeze dryer (Labconco, Kansas, MO, USA) [22]. Crude extracts obtained had an appearance of a yellow light powder with a yield of 22.0% *w*/*w*. To be used in experiments, the extract was dissolved in distilled water and filtered using a 0.22 μm Millipore filter membrane. 

### 2.4. Gas Chromatography-Mass Spectrometry Analysis (GC-MS)

The *LM* extract was dissolved in Milli-Q water and filtrated through a 0.22 µm filter, and then diluted 1:1 in methanol and submitted to GC-MS analysis, without any further purification. The GC-MS system (Hewlett-Packard, San Diego, CA, USA) consisted of a GC, series 6890, interfaced with an MS detector (model 5973). GC-MS analysis was performed using an HP-5ms column (cross-linked methyl 5% phenyl silicone, 30 m × 0.25 mm i.d., 0.25 µm film thickness). The column oven temperature was initially held at 50 °C for 2 min, then programmed to reach 230 °C, at a rate of 20 °C/min and held there for 6 min, and then increased by 1 °C/min until 240°C and held there for 5 min. The total run time was 32 min. The temperature of the injector port and of the interface were both set to 270 °C. The carrier gas (helium) flow rate was 1 mL/min. The ionization energy was set at 70 eV. Mass spectra were collected by scanning from m/z 50 to m/z 700 at 20 Hz. For tentative identification, the Wiley 275 mass spectral library was employed.

### 2.5. High Performance Liquid Chromatography-High Resolution Mass Spectrometry Analysis (HPLC-HRMS) 

*LM* extract was dissolved in Milli-Q water and filtrated through a 0.22 µm filter before submitting to HPLC-HRMS analysis, without any further purification. HPLC analysis was performed using an Agilent 1100 system equipped with a Discovery 150 × 4.6 mm reversed phase C18 column. The mobile phase was composed of water with 0.1% formic acid (A), and acetonitrile (B). A stepwise gradient was used starting with 95% (A): 5% (B), and held there for 5 min, changed to 80% (B) in 40 min and then return to initial conditions 95% (A): 5% (B) in 45 min, with a flow of 0.8 mL/min. For the HRMS detection, a Bruker’s MaXis Impact ESI Q-TOF mass spectrometer with Sodium formate (positive) as calibrant (positive scanning mode *m*/*z* 50-1500) was used. The UV detection was done using an Agilent 1100 series Diode Array Detector (DAD) (Agilent Technologies, Palo Alto, CA, USA).

### 2.6. Oral Glucose Tolerance Test (OGTT)

GK and W rats (*n* = 6 per group), fasted for 10–12 h, received an oral single administration of *LM* extract (2000, 1000 and 500 mg/kg b.w.), dissolved in 2 mL distilled water, one hour before the OGTT. The evaluation started with the oral glucose challenge, 2 g/kg b.w. for GK rats and 3 g/kg b.w. for W rats. Blood samples were collected, from the tip of the tail, immediately after the glucose administration (time 0), 30, 60, 90, and 120 min. Glycemia was measured in every time point with a glucometer Accu-check Aviva (Roche Diagnostic GmbH, Indianapolis, IN, USA). Serum insulin levels were measured at time 0 and 30 min by radioimmunoassay (RIA). The placebo groups for both GK and W rats received the administration of the extract vehicle, i.e., distilled water [22,23,24].

### 2.7. Pancreatic Islets Isolation

Pancreatic islets isolation was performed as described previously [22,24,25]. Briefly, collagenase solution (2.4 mg/mL for GK rats and 0.9 mg/mL for W rats) (Sigma-Aldrich, St. Louis, MO, USA) was injected through the bile duct to insufflate the pancreas, then tissue was collected and digested in a water bath for 24 min at 37 °C. Islets were separated by density-gradient centrifugation using a mixture of Histopaque 1119 and 1077 and were hand-picked using a stereomicroscope. Isolated islets were cultured overnight at 37 °C, with an atmosphere of 5% CO_2_-95% air in RPMI 1640 supplemented with 30 mg l-glutamine, 11 mM glucose, antibiotics (100 IU/mL penicillin and 0.1 mg/mL streptomycin) (Invitrogen, Carlsbad CA, USA) and heat-inactivated fetal calf serum (10%).

### 2.8. Cytotoxicity

Cellular toxicity was determined by MTT assay [26,27] evaluated in batches of W islets treated with *LM* extracts (5–20 mg/mL) in supplemented RPMI 1640 medium (30 mg l-glutamine, 11 mM glucose, 100 IU/mL penicillin, 0.1 mg/mL streptomycin and heat-inactivated fetal calf serum (10%), during 1, 8 and 24 h at 37 °C. 

### 2.9. Islet Insulin Release

Overnight cultured islets were pre-incubated in Krebs-Ringer bicarbonate (KRB) buffer with 3.3 mM glucose during 30–45 min at 37 °C. Then, batches of 3 islets of similar size from GK and W rats were incubated in low (3.3 mM) or high (16.7 mM) glucose KRB, with or without *LM* extract (5–20 mg/mL) during 60 min at 37 °C in a shaking water bath [22,25]. After incubation, 200 μL of each condition were collected for insulin quantification by RIA assay [23,24,25].

### 2.10. Islet Perifusion 

Batches of 40 or 50 islets from GK or W rat were layered between polystyrene beads (Bio-Rad Laboratories, Inc., Hercules, CA, USA) and were perifused continuously using a peristaltic pump (Ismatec SA, Zurich, Switzerland) in a perifusion chamber. KRB buffer with 3.3 mM glucose was perifused during the first 20 min (−20 to min 0), to establish the basal insulin secretion rate then, the buffer was changed to KRB 3.3 mM glucose plus *LM* extract (10 mg/mL), from time 0 to 14 min, and to KRB 16.7 mM glucose plus *LM* extract (10 mg/mL), from time 16 to 30 min; finally, KRB buffer was switched back to 3.3 mM glucose without *LM* extract, for the last 20 min [25]. Perifusion buffer fractions were collected every second minute for insulin quantification by RIA assay. The AUC in presence of *LM* was calculated for periods of *LM* treatment in low glucose, period 0 to 14 min and high glucose, period 16 to 30 min, subtracting the basal value at the beginning of each treatment and was compared to AUC of the same periods of untreated islets [22].

### 2.11. Glucose Uptake Evaluation

Experiments were performed as described before [28,29]. Briefly, adipocytes from GK and W rats were isolated from rat epididymal by digestion with type II collagenase 0.25 mg/mL (Sigma-Aldrich) during 120 min at 37 °C and filtered through a coarse nylon mesh (250 μm) in a Krebs–Ringer medium (139 mM NaCl, 5.4 mM KCl, 1 mM NaH_2_PO_4_, 1 mM MgSO_4_, 2.2 mM CaCl_2_, pH 7.4), 20 mM Hepes buffered, containing 2% of bovine serum albumin with 7 mM glucose. After isolation, 1% adipocyte suspension was incubated for 2 h at 37 °C with [3-^3^H]-glucose (1 μCi/mL, Perkin Elmer), 1 mM d-glucose solution (Sigma Aldrich) and *LM* hydroethanolic extract in a range of concentrations from 5–20 mg/mL. After the incubation, the vials were transferred into ice to stop the reactions and 3 mL of scintillation cocktail (2 M PPO (2,5-diphenyloxazole) and 0.02 M POPOP (1,4-Bis(4-methyl-5-phenyl-2-oxazolylbenzene))), dissolved in toluene (Sigma Aldrich) was added. A liquid Scintillator Analyzer (Tri-Carb 1900TR, Packard, Detroit, MI, USA) was used to measure the radioactivity of ^3^H-glucose incorporated in the de novo synthesized lipids since is proportional to ^3^H-glucose taken up by the cells. Insulin concentrations from 0.1 to 172 nM used as a control of glucose uptake.

### 2.12. Sub-Acute Oral Toxicity

The potential sub-acute oral toxicity (28 days) of *LM* extract was evaluated in W rats following the guideline 407 set by the Organization for Economic Cooperation and Development (OECD) [30]. *LM* extract was incorporated to the regular food in a proportion to achieve a daily dose of 1000 mg/kg b.w. Changes in skin, fur, eyes, the occurrence of secretions, lacrimation, piloerection were monitored. At the endpoint, blood samples were collected to determine the hematological and serum biochemical parameters [22,31].

### 2.13. Long-Term Treatment Evaluation

The effect of long-term treatment of *LM* was performed by daily administration for 21 days both in GK and W rats (*n* = 6 per group): Group 1: GK rats treated with *LM* 1000 mg/kg b.w.; group 2: GK rats, treated with vehicle, distilled water; group 3: W rats treated with *LM* 1000 mg/kg b.w.; group 4: W rats treated with vehicle, distilled water. The body weight and non-fasting glucose levels were measured every third day and the OGTT was performed on days 0, 10 and 20. Blood samples were collected to measure serum insulin by RIA and plasma glycated hemoglobin (HbA1c) by ELISA commercial kit (Cat. No. 80300, Crystal Chem INC, Zaandam, The Netherlands). After 21 days, pancreatic tissue was collected to isolate pancreatic islets for evaluating the insulin release [22]. 

### 2.14. Mechanisms of LM-Dependent Insulin Release 

To elucidate the mechanism by which *LM* stimulates insulin release, GK and W rat islets were treated with *LM* (10 mg/mL) in presence of different compounds that block specific points of the insulin secretion pathway. To evaluate whether *LM* exerts its effect by the closure of the adenosine triphosphate (ATP) sensitive potassium channels (K-ATP), 0.25 Mm diazoxide (DX) (Sigma-Aldrich, St. Louis, MO, USA), an opener of K-ATP channels was used. To study whether the *LM* effect is dependent of depolarization membrane events, islets were incubated with 50 mM KCl, (Sigma-Aldrich, St. Louis, MO, USA), to depolarize the β-cells, plus 0.25 mM DX to keep K-ATP channels opened. To assess the role of L-type Ca^2+^ channels on *LM* effect, 10 µM nifedipine (NF), (Sigma-Aldrich, St. Louis, MO, USA), an inhibitor of L-type Ca^2+^ channels, was used. To study the role of protein kinase A (PKA) and protein kinase C (PKC) on *LM* effect, islets were incubated with 10 μM *N*-[2-bromocinnamylamino)ethyl]-5-isoquinolone sulfonamide (H89), a PKA-inhibitor, or 1.5 μM calphostin-C (Cal-C), a PKC inhibitor. Finally, to explore whether *LM* promotes insulin release by exocytotic G proteins, islets were pretreated at 37 °C overnight with 100ng/mL pertussis toxin (PTx), an inhibitor of G proteins, in complete RPMI 1640 culture medium (SVA, Sweden) (11 mM glucose, 30 mg l-glutamine, 10% heat-inactivated fetal calf serum, and antibiotics (100 IU/mL penicillin and 0.1 mg/mL streptomycin, Invitrogen, Carlsbad, CA, USA). After PTx exposure, islets were incubated in presence or absence of *LM* (10 mg/mL) in KRB buffer 3.3 mM and 16.7 mM glucose. For all the treatments, 200 μL aliquot of KRB medium were collected for insulin quantification by RIA [22,24,32].

### 2.15. Statistical Analysis

Results are presented as mean ± standard error of mean (SEM). In several instances, control or placebo results have been published in a previous study on another plant, *Amaranthus caudatus* [22], since studies with *Amaranthus caudatus* and *Lupinus mutabilis* (present study) were performed in parallel. Statistical significance, P value of less than 0.05, was analyzed using two-way analysis of variance (ANOVA) for OGTT, serum insulin, glycated hemoglobin, and insulin release whereas paired Student’s t-test was used for AUC analysis. Bonferoni’s Post Hoc Test was used for correction of multiple testing. Data were analyzed using Graph Pad Prism Software (version 6.0, GraphPad Software, San Diego, CA, USA). 

## 3. Results

### 3.1. LM Extract Constituents 

**Figure 1:**
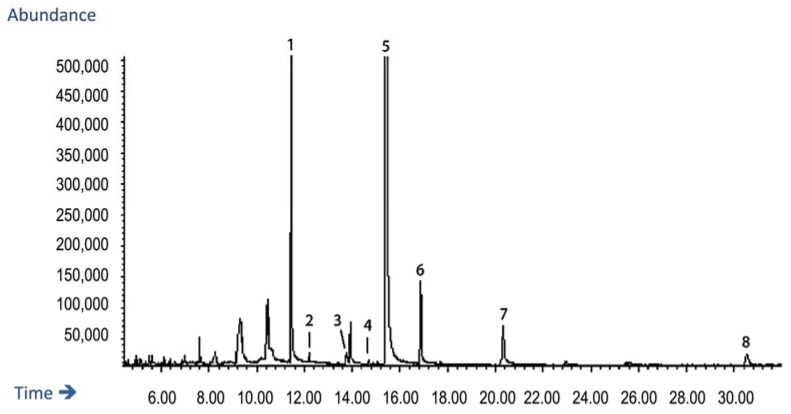
GC-MS total ion current (TIC) chromatogram of the *Lupinus mutabilis* (*LM)* extract. From the MS spectra, the fragmentation patterns of the eluting peaks were compared against the Wiley 275 mass spectral library, and the following compounds were tentatively assigned: Peak 1 Sparteine MW 234; peak 2 Palmitic acid MW 256; peak 3 Linoleic acid and Oleic acid MW 280 and 282 respectively; peak 4 and 5 Lupanine/α-Lupanine MW 248; peak 6 Nuttalline MW 264; peak 7 Oxylupanine MW 264, and peak 8 11,12-Dehydrolupanine MW 246.

GC-MS analysis was performed to identify volatile components of the *LM* extract. The GC-MS total ion current (TIC) chromatogram is shown in (Figure 1). The fragmentation pattern of the eluting peaks was compared against the Wiley 275 mass spectral library, which indicated that *LM* extract could tentatively be assigned to contain sparteine, palmitic acid, linoleic acid, oleic acid, lupanine, nuttalline, oxylupanine, and 11,12-dehydrolupanine.

**Figure 2:**
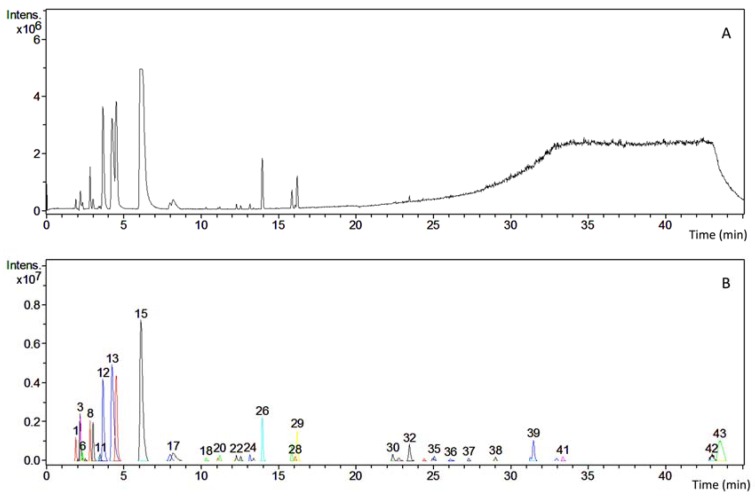
The *LM* extract consists of a complex mixture of phytochemicals. An HPLC chromatogram with (**A**) total ion chromatogram TIC and (**B**) processed chromatogram showing the presence of at least 43 different peaks each corresponding to a compound with a discrete molecular weight. From the retention time and protonated molecular ions [M + H]^+^ from the MS spectra, some peaks corresponding to QA were tentatively assigned: Peak 12 and 14 Hydroxylupanine/Nuttalline/Oxylupanine MW 264; Peak 13 Sparteine MW 234; Peak 15 and 17 Lupanine/α-Lupanine MW 248.

HPLC-HRMS analysis was performed to identify non-volatile components of *LM* extract. More than 43 different types of phytochemicals (Figure 2) were present including for examples nuttalline, sparteine, lupanine. Due to the complexity of the chromatogram no further attempt was made to identify the chemical composition of the phytochemicals constituting the extract.

### 3.2. LM Improves Glucose Tolerance in GK and W Rats by Increasing Serum Insulin Levels in a Glucose-Independent Manner

**Figure 3:**
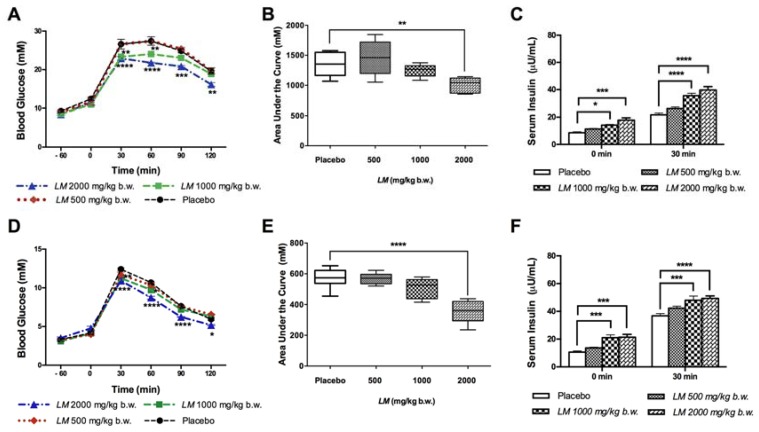
*LM* improves Glucose Tolerance and increases serum insulin. *LM* effect on glucose tolerance was evaluated in 12 h fasted, animals received a single oral administration of *LM* extract (500–2000 mg/kg b.w.) one hour before glucose-challenge. Blood glucose was determined at 0, 30, 60, 90 and 120 min; Goto-Kakizaki (GK) rats (**A**) and Wistar (W) rats (**D**). The area under the curve (AUC) was calculated from time 0 to 120 min in GK rats (**B**) and W rats (**E**). Serum insulin during the OGTT was determined at 0 and 30 min in GK rats **(C**) and W rats (**F**). Data are presented as means ± standard error of the mean (SEM) (*n* = 6). * *p* < 0.05, ** *p* < 0.01, *** *p* < 0.001, **** *p* < 0.0001 when compared to placebo group

The improvement of glucose tolerance by *LM* (2000 mg/kg b.w.) in GK rats started 30 min after glucose administration and continued during the following time points (Figure 3A). *LM* effect was dose-dependent, where 1000 mg/kg b.w. improved glucose tolerance at early time points tested, no significant effect was found with the lowest dose tested (500 mg/mL). *LM* effect during the OGTT was reflected also on the AUC of glucose that was reduced in GK rats at 2000 mg/kg b.w. to 1005.0 ± 57.6 mM/120 min compared to placebo GK rats (1357.0 ± 91.6 mM/120 min) (Figure 3B). Serum insulin levels measured during the OGTT were higher already in time zero, i.e., a time point that corresponds to 60 min post *LM* administration; 1.65-fold (1000 mg/kg b.w.) and 2.08-fold (2000 mg/kg b.w.) compared to placebo GK rats. Serum insulin continued to increase after 30 min of glucose challenge 1.64-fold and 1.83-fold (2000 and 1000 mg/kg b.w., respectively) (Figure 3C). 

A similar effect was found in W rats, where *LM* (2000 mg/kg b.w.) improved glucose tolerance during the OGTT starting at 30 min with a lower effect of *LM* (1000 mg/kg b.w.) but not of 500 mg/kg b.w. (Figure 3D). The AUC of glucose of *LM* (2000 mg/kg b.w.) treatment was reduced to 353.8 ± 31.3 mM/120 min, versus placebo W rats (572.0 ± 27.6 mM/120 min) (Figure 3E). Serum insulin levels in W rats were also higher, 60 min after *LM* administration (1000 and 2000 mg/kg b.w.) and 30 min after glucose challenge (1000 mg/kg b.w. and 2000 mg/kg) (Figure 3F).

### 3.3. LM Stimulates The in vitro Insulin Release Independent of Glucose

**Figure 4:**
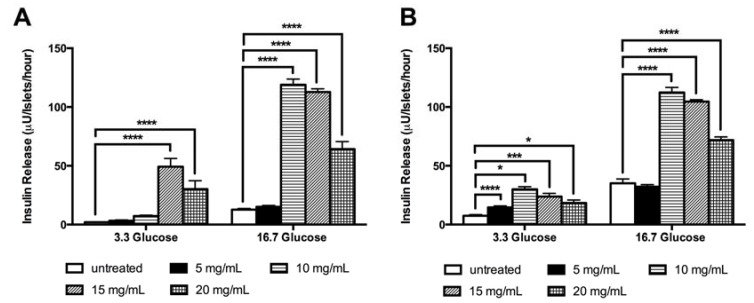
*LM* induces *in vitro* insulin release in batch incubated islets. The insulin release was evaluated in batch incubated GK rat islets (**A**) and W rat islets (**B**) incubated with low (3.3 mM) and high (16.7 mM) glucose in presence of *LM* (5–20 mg/mL). Insulin concentration was measured by RIA. The data are presented as means ± standard error of the mean SEM (*n* = 8). * *p* < 0.05, *** *p* < 0.001, **** *p* < 0.0001 when compared to untreated islets.

*LM* stimulated the *in vitro* insulin release in GK rat islets in both low and high glucose concentrations; the effect was observed with all doses tested and interestingly, the highest stimulatory effect was observed using the lowest *LM* concentration. *LM*, in low glucose condition (3.3 mM), stimulated insulin release in GK rat islets 6.6-fold (15 mg/mL) and 4.0-fold (20 mg/mL); and in high glucose condition (16.7 mM) 9.4-fold (10 mg/mL), 8.9-fold (15 mg/mL) and 5.0-fold increase (20 mg/mL) compared to untreated islets (Figure 4A). 

*LM* effect on insulin release, in high glucose conditions, was comparable with the β-cell secretagogue drug glibenclamide (Supplementary Material Figure S1). In W rats islets, *LM* effects were found with all concentrations tested in low glucose (5–20 mg/mL) and in high glucose (10–20 mg/mL) (Figure 4B). No cytotoxic effect in batch islets cultured in presence of *LM* (5–20 mg/mL) was observed after 1 h of exposure. After 8 and 24 h of culture the highest concentration (20 mg/mL) reduced viability by 10% and 19%, respectively (Supplementary Material Figure S2). 

### 3.4. The LM Effect on The Kinetics of Insulin Release is Glucose Independent. 

**Figure 5:**
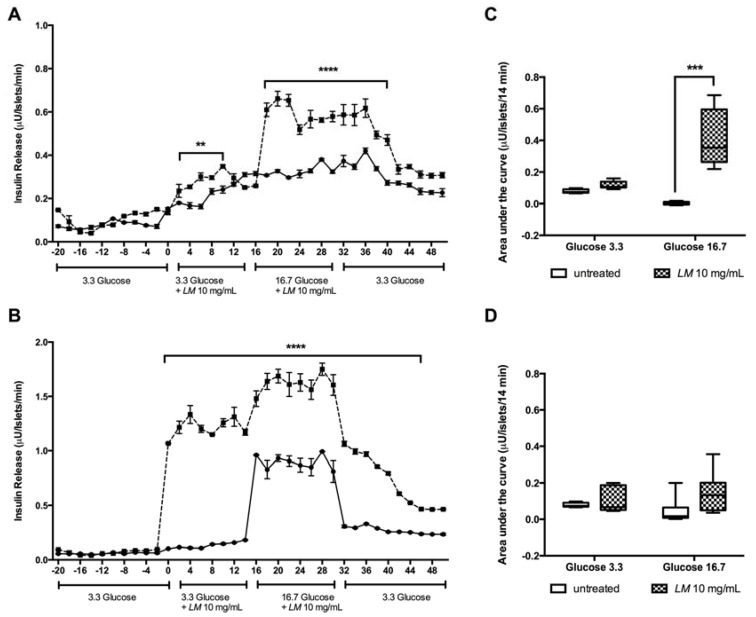
*LM* induces *in vitro* insulin release in perifused islets. Batches of 40–50 islets from GK (**A**) and W rat (**B**) were perifused with low glucose (3.3 mM), from time 0 to 14 min, and with high glucose (16.7 mM), from time 16 to 30 min, in presence ---◾--- or absence –•– of *LM* (10 mg/mL). The AUC of the insulin release from the intervals in low and high glucose, in the presence or absence of *LM* were calculated; GK rat (**C**) and W rat (**D**) islets. Data are presented as means ± standard error of the mean SEM (*n* = 4). ** *p* < 0.01, *** *p* < 0.001, **** *p* < 0.0001 when compared to untreated islets.

The effect on insulin secretion was monitored in islets perifused with *LM* (10 mg/mL). Significant differences in insulin release were observed when GK rat islets were perifused during the period of 0–10 min with *LM* in low glucose (3.3 mM) and during the period of 18–30 min, when islets were perifused with *LM* in high glucose (16.7 mM) compared to untreated perifused islets during the respective time points (Figure 5A). Interestingly, the effect on insulin release continued after of *LM* removal, being statistical significant up to 42 min. A similar pattern was found in W rat islets perifused with *LM* during the periods of 0–14 min (3.3 mM) and 18–30 min (16.7 mM) (Figure 5B).

The stimulatory effect of *LM* was, however, less pronounced when the AUC of the insulin release was calculated. In GK rat islets perifused in low glucose (3.3 mM), differences were no significant while in high glucose (16.7 mM) the AUC was increased 95.3-fold (Figure 5C). In W rats, no significant differences in the AUC of insulin release were found either in low or high glucose conditions (Figure 5D).

### 3.5. LM Does not Stimulates the in vitro Glucose Uptake

The *LM* effect on insulin resistance was evaluated by a glucose uptake assay measured in adipocytes isolated from epidydimal fat from GK and W rats but no significant effect was detected (Supplementary Material Figure S3).

### 3.6. The Anti-Diabetic Effect of the Oral Long-Term LM Treatment is Mediated by the Increase of Insulin Release

The long-term oral treatment with *LM* (1000 mg/kg b.w.) improved the glucose tolerance in GK rats starting from day 10 (90 min of the OGTT) and after day 20 (30–120 min of the OGTT) (Table 1). In W rats *LM* improved the glucose tolerance in both day 10 (30–60 min of the OGTT) and day 20 (30–120 min of the OGTT) (Table 1).

The improvement of glucose tolerance was also reflected in the AUC of glucose in GK rats at day 20 (Table 1). Similarly, in W rats, *LM* reduced the AUC of glucose at day 20 (Table 1). *LM* treatment did not show significant differences in body weight gaining compared to placebo group (Supplementary Material Figure S4). 

**Figure 6:**
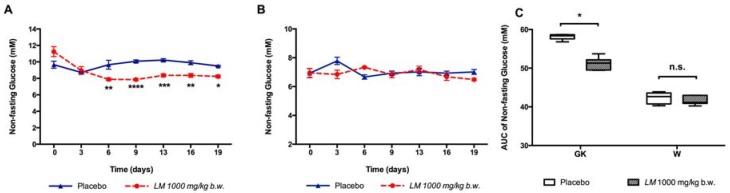
*LM* long-term treatment reduces the non-fasting glucose. The non-fasting glucose was determined every third day in GK rats (**A**) and in W rats (**B**). The AUC for each group was calculated from day 0 to day 19 (**C**). Data are presented as means ± standard error of the mean SEM (*n* = 6). * *p* < 0.05, ** *p* < 0.01, *** *p* < 0.001, **** *p* < 0.0001 when compared to Placebo.

*LM* treatment reduced the non-fasting glucose levels, measured every third day from day 6 to day 19 in GK rats (Figure 6A), but not in W rats (Figure 6B). The AUC of non-fasting glucose was reduced in GK rats (51.2 ± 0.6 mM vs. 58.2 ± 0.3 mM) (Figure 6C), but not in W rats. 

**Figure 7:**
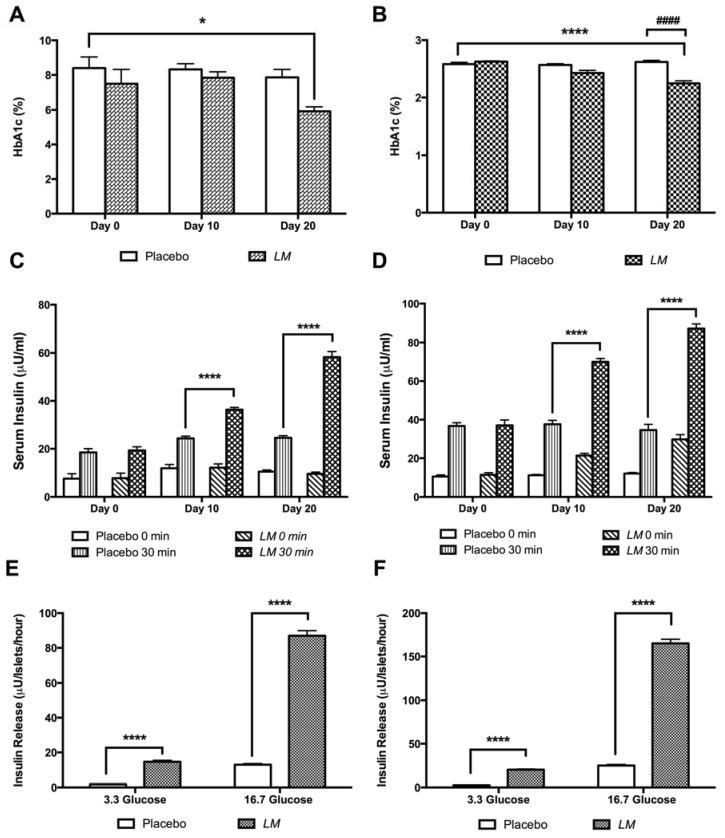
*LM* long-term treatment reduces plasma HbA1c, increases serum insulin and improves insulin release. Blood samples were collected during the OGTT performed on day 0, 10 and 20 to measure plasma HbA1c in GK rats (**A**) and in W rats (**B**) (*n* = 6). Serum insulin was measured at 0 and 30 min during the OGTT; GK rats (**C**) and W rats (**D**) (*n* = 6). Pancreatic islets isolated from GK rats (**E**) and Wistar rats (**F**) at the end point of treatment were incubated in low (3.3 mM) and high (16.7 mM) glucose. Insulin concentration was measured by RIA. The data are presented as means ± SE, * *p* < 0.05, ** *p* < 0.01, *** *p* < 0.001, **** *p* < 0.0001 when compared to placebo group and ^####^ *p* < 0.0001 when compared to same group values at different days of treatment.

Plasma HbA1c levels were reduced by treatment with *LM* both in GK rats (5.9 ± 0.3%) at day 20 compared with placebo GK rats at day zero (8.4 ± 0.8%) (Figure 7A), and in W rats (2.2 ± 0.04%) compared to placebo W rats, at day zero (2.6 ± 0.03%) (Figure 7B). Serum insulin increased in GK rats 1.5-fold at day 10 and 2.4-fold at day 20 (Figure 7C), and in W rats it increased 1.9-fold at day 10 and 2.5-fold at day 20 (Figure 7D). 

Furthermore, *LM* showed a direct effect on insulin secretion in islets isolated from treated animals (day 21). In GK rats, in vitro insulin release increased 8.1-fold in low glucose (3.3 mM) and 6.7-fold in high glucose condition (16.7 mM) compared to islets isolated from the placebo group (Figure 7E). A similar effect was found in islets isolated from *LM* treated W rats, where insulin release increased 8.0-fold in low glucose (3.3 mM glucose), and 6.6-fold in high glucose (16.7 mM glucose) (Figure 7F).

Results of the *LM* sub-acute toxicity evaluation did not show significant differences in body weight and (Supplementary Material Figure S5) among the hematological indicators i.e., red and white blood cells number, hematocrit and hemoglobin, and serum biochemical parameters i.e., aspartate and alanine transaminase, alkaline phosphatase and uric acid between *LM* treated and placebo W rats (Supplementary Material Table S1).

### 3.7. LM-Dependent Insulin Release is Mediated by L-Type Calcium Channel, PKC and PKA Systems, and Exocytosis by G-Proteins

**Figure 8:**
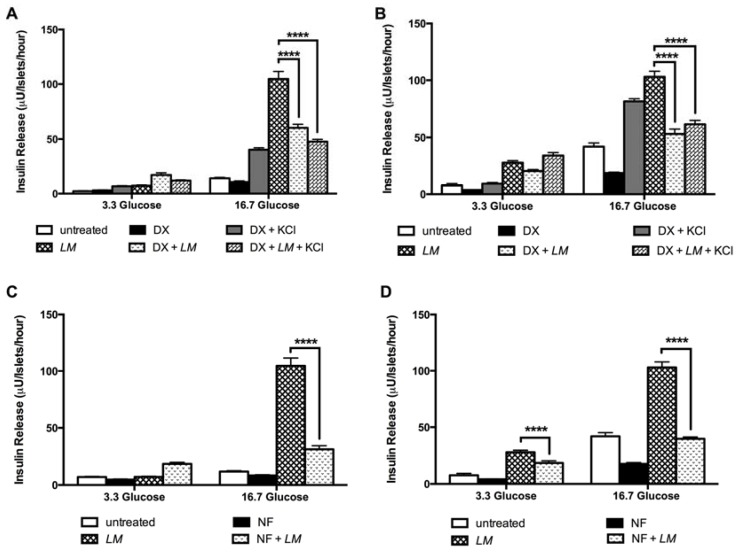
*LM*-dependent insulin release is mediated by ATP sensitive potassium channels and L-type calcium channels. *LM* effect was evaluated in islets cultured in low (3.3 mM) and high (16.7 mM) glucose in presence of DX (0.25 mM) and or KCl in GK (**A**) and W rats islets (**B**); NF (10uM) in GK (**C**) and W rats islets (**D**). Insulin concentration was measured by RIA. Data are presented as means ± standard error of the mean (SEM) (*n* = 8). **** *p* < 0.0001 when compared to islets treated with *LM* alone.

Diazoxide (DX) a selective ATP-sensitive K^+^ channel opener inhibited the insulin release-dependent of LM in GK rat islets by 42% and 48.5% in W rat islets (16.7 mM). In islets incubated with high concentrations of K+, to induce transient insulin release, DX inhibited the LM-dependent insulin release by 54% compared to the value of LM alone (Figure 8A). In W rat islets exposed to conditions of high (K^+^) plus DX, 40.2 % of inhibition was observed (Figure 8B).

To evaluate the role of L-type voltage-dependent Ca^2+^ channels in *LM* effect we used nifedipine (NF) that reduced the insulin release by 70% in GK islets in 16.7 mM glucose (Figure 8C) and in W rat islets by 34.5% in 3.3 mM glucose and 61.4% in 16.7 mM glucose conditions (Figure 8D).

**Figure 9:**
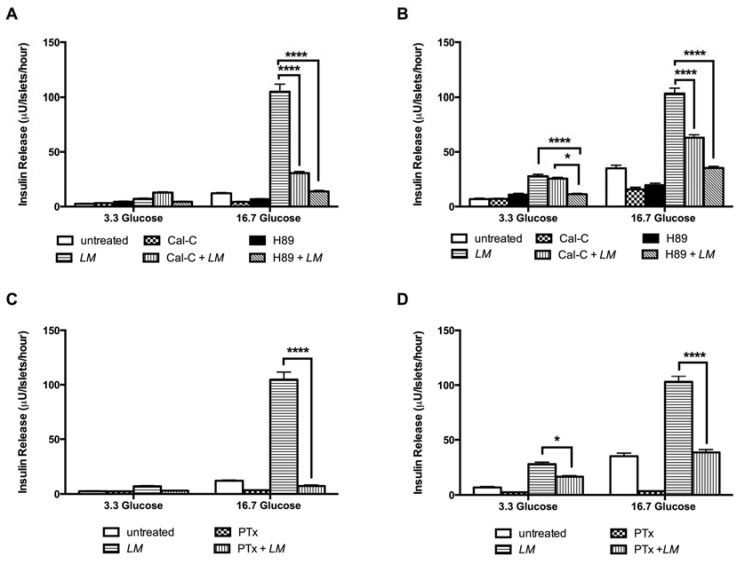
*LM* effect on insulin release is mediated PKA and PKC systems and G protein-coupled exocytosis. *LM* effect was evaluated in islets cultured in low (3.3 mM) and high (16.7 mM) glucose in presence of Cal-C (1.5 uM) or H89 (10 uM) in GK (**A**) and W rats islets (**B**); and PTx (100 ng/mL) in GK (**C**) and W rats islets (**D**). Insulin concentration was measured by RIA. Data are presented as means ± standard error of the mean (SEM) (*n* = 8), of triplicates from four independent experiments. * *p* < 0.05, **** *p* < 0.0001 when compared to islets treated with *LM* alone.

To explore whether *LM* stimulated insulin release independently of membrane depolarization, i.e., the K-ATP-independent pathway, we used dihydrochloride hydrate (H89), a selective potent cell permeable inhibitor of cAMP-dependent protein kinase (PKA) and calphostin C (Cal-C), a protein kinase C inhibitor (PKC). *LM* effect on insulin release was reduced 86.7% by H89 and 70.8% by Cal-C in GK rat islets only in high glucose conditions (Figure 9A). In W rat islets, *LM* effect was reduced 59.2% by H89 and 7.7% by Cal-C in low glucose and 66% by H89 and 39% by Cal-C in high glucose condition (Figure 9B).

Finally, pertussis toxin (PTx) was used to explore the effect of *LM* on the insulin exocytosis. In GK rat islets PTx inhibition was 93% (16.7 mM glucose) (Figure 9C), while in W rat islets PTx inhibition was 40% in low glucose and 63% in high glucose (Figure 9D).

## 4. Discussion

In the present study, we found that *LM* seed hydroethanolic extract improved glucose tolerance in type 2 diabetic GK rats and in healthy W rats by enhancing insulin release. This effect was mainly mediated by L-type calcium channel, the PKC and PKA systems and G protein-coupled insulin exocytosis, and partially by K-ATP channels of the β-cells. Since control of the glycemia in diabetes is important and helps to avoid the development of diabetes complications, *LM* hydroethanolic extract might be a promising nutraceutical product to restore glycemic homeostasis in the context of T2DM.

A single oral treatment with *LM* improved glucose tolerance and increased serum insulin levels in the diabetic GK rats as well as in the healthy W rats. In both types of rats, the effect was dose-dependent and the reduction in serum glucose was gradual and did not produce a hypoglycemic state since glucose values at the end of the OGTT were not lower than the fasting initial glucose values. Those findings support the traditional use that advises drinking the water where *LM* seeds were washed, just after meals, in order to control the glycemia levels. Based on those results we can infer that the acute *LM* mechanism of glucose lowering is a non-glucose-dependent insulin secretion and that *LM* components have a rapid absorption to reach bloodstream and then the target organ, the pancreas. 

In batch-incubated islets, *LM* effect on insulin release was glucose-independent, in GK rats when using high concentrations, while W rats islets appear to be more sensitive to *LM* effect since insulin release was augmented in all tested concentrations. Interestingly, the highest *LM* concentrations tested showed less insulin-releasing effect; this pattern could be explained by the possible interaction of inhibitory compounds that might be present in the crude extract or could be attributed to the toxicity of the alkaloid content at that specific dose [33,34], however *in vitro* toxicity results showed that used *LM* concentrations had no toxic effects in short periods of exposure. In further experiments, we used *LM* concentration (10 mg/mL) that did not increase insulin release in low glucose conditions. *LM* also improved the insulin release in perifused islets of GK; in low glucose condition it was partially but not significantly augmented while in high glucose was further augmented. When *LM* was removed, insulin secretion gradually returned to basal levels, proving that *LM* does not have a toxic effect on β-cells. In W rats even though insulin release increased during *LM* perifusion, in a similar pattern as in GK rats, it did not reach significance when the AUC was calculated. Furthermore, *LM* effect was even higher than the one observed by the insulin secretagogue drug glibenclamide but as we explained before, in animal experiments *LM* effect showed to be transient, and in perifused islets the insulin release basal levels were restored gradually when *LM* was removed, meaning that the *LM* effect does not induce a hypoglycemic state.

*LM* long-term treatment restored glucose metabolism as evidenced by the reduction of the non-fasting glucose levels in GK rats but this effect was not observed in W rats. *LM* extract seemed to control variable high glucose levels present only in GK rats, abnormal glucose levels that are not present in W rats, it seems that a sustainable *LM* effect needs to be glucose-dependent, as in vitro studies showed. On the contrary, *LM* long-term treatment reduced the HbA1c, in both GK and W rats, after 20 days, and the increased levels of serum insulin in treated animals explained this effect. Moreover, *LM* effect was confirmed by the improvement of insulin release in pancreatic islets isolated from treated animals, suggesting that *LM* controls glycemia promotion of β-cell function and insulin release, results that support the daily consumption of components present in *LM* extract in order to control hyperglycemia in diabetic patients. On the other hand, *LM* long-term treatment did not have an impact on body weight and in sub-acute toxicity experiments, *LM* conditions have proven to be safe.

*LM* contains 42% of protein and 18% fat on average; its proteins are rich in the essential amino acid lysine [14,35,36]. Besides, *LM* seeds contain high amounts of alkaloids, molecules responsible for its bitter taste [14,37]. Regarding its glucose-lowering effect, literature reports that cooked *LM* seeds reduce glycemia in healthy and slightly hyperglycemic volunteers [16] and its purified alkaloids reduce glycemia in type 2 diabetes patients [17]. Our results of the GC-MS and HLPC-HRMS analysis showed that *LM* extract is rich in alkaloids as sparteine, lupanine, nuttalline, oxylupanine, and 11,12-dehydrolupanine and other phytochemicals as palmitic acid, linoleic acid, and oleic acid, composition similar to what was described for other Lupinus genus species [14,17,38,39]. Thus, our findings could be explained by the alkaloid content found in the *LM* crude extract. 

To study the mechanism of *LM* insulin-releasing effect, different blockers of proteins of the insulin-releasing cascade were used [22,32]. In the normal glucose metabolism, glucose is transported into pancreatic β-cell by glucose transporter 2 (GLUT2), where is metabolized via glycolysis and then oxidized by Krebs cycle to produce ATP [40,41], resulting in an increase of ATP/ADP ratio. This, in turn, induces the closure of potassium ATP-sensitive (K-ATP) channels to depolarize the β-cell membrane. The depolarization activates the voltage-dependent calcium (L-type Ca^2+^) channels leading the entrance of calcium [42,43] that finally stimulates insulin release. 

To evaluate the role of K-ATP channels on *LM* effect, diazoxide (DX) was used in order to maintain K-ATP channels opened, whereby the *LM* effect in both GK and W rat islets was partially reduced at 16.7 mM glucose. Therefore, the *LM* effect seems to depend on the closure of K-ATP channels. Moreover, the *LM* effect was inhibited in the presence of diazoxide and high concentrations of KCl, to depolarize β-cell membranes. 

Nifedipine (NF), an L-type Ca^2+^ channel blocker [25,32], was used to evaluate the involvement of L-type Ca^2+^ channels on the *LM* effect. In GK islets incubated in presence of NF, the *LM* effect on insulin release was reduced at 16.7 mM glucose, while in W islets the *LM* effect was partially reduced at both low and high glucose. Thus, the *LM* effect is also mediated by the activation of L-type Ca^2+^ channels.

To explore the participation of PKA and PKC activation in *LM* effect, the PKA inhibitor, H89, and the PKC inhibitor, calphostin-C were used. Both inhibitors reduced the *LM* effect, in GK and W islets, with strong suppressing effect in GK islets. These findings suggest that *LM* effect on β-cells involves the activation of both the PKA and PKC systems. The glucose-induced insulin release can be modulated by intracellular signals via second messengers, such as cyclic AMP (cAMP) and diacylglycerol (DAG), that can induce insulin release through PKA and PKC activation, respectively [44]. Further evaluation of the significance of those second messengers should be made. 

Pertussis toxin, an inhibitor of G-proteins via ADP-ribosylation, was used to evaluate the role of Guanine nucleotide-binding proteins (G-proteins) on *LM* effect. PTx inhibited the *LM*-dependent insulin release in both GK and W islets, although this effect was greater in GK islets than in W islets. Therefore, it appears that the *LM* effect involves the activation of G-proteins, so-called Ge-proteins, associated with the exocytosis of insulin [25]. Alternatively, Gq-protein activates PLC and Gs-protein stimulates the synthesis of cAMP, a second messenger that finally activates PKC and PKA. Thus, *LM* could also activate the synthesis of those second messengers via G proteins and thereby finally enhance the insulin release [45]. 

Several studies of Lupinus species were reported to have a biological effect due to its alkaloid content. A stimulatory effect on insulin release is reported for sparteine, lupanine and its 13-α-hydroxy- or 17-oxo-derivative as well as for the synthetic derivative 2-thionosparteine [44] and Diazoxide (0.1 mM) decreases the effect of all Lupinus alkaloids, without a complete suppression [38,40,41,46], findings congruent with our results. Consequently, according to our results, the blockage of β-cell K-ATP channels is at least one of the mechanisms involved in the secretagogue effect of *LM*. It is important to highlight that low concentrations of the alkaloids need to be used to avoid any side effects due to the toxicity of quinolizidine alkaloids. Lupin alkaloids are mainly neurotoxins that affect nicotinic and muscarinic acetylcholine receptors and Na^+^ and K^+^ channels [40].

However, the interaction or modulation of other subsequent steps involved in glucose-induced insulin secretion, i.e., the opening of voltage-dependent Ca^2+^ channels triggering exocytosis of insulin-containing granules or non-glucose-induced insulin secretion, by *LM* alkaloids or other components present in *LM* hydroethanolic extract, was not reported before. 

Ca^2+^ intracellular increase in β-cell is related to activation of kinases, such as PKA and PKC that are responsible for the phosphorylation of proteins able to modulate insulin exocytosis, events that could be indirectly inhibited by NF. Additionally, Ca^2+^ activates the receptor-coupled enzyme phospholipase C (PLC), and its activation hydrolyzes the plasma membrane phospholipid phosphatidylinositol bisphosphate (PIP2) into the second messengers, diacylglycerol (DAG) and inositol trisphosphate (IP3). DAG activates PKC, and IP3 liberates Ca^2+^ from the endoplasmic reticulum [44,45,47]. Hence, further experiments to analyze events related to protein kinase activation mediated by Ca^2+^ and the second messengers, DAG and IP3 in presence of *LM* need to be made. 

## 5. Conclusions

Our study provides evidence of the *LM* anti-diabetic effect through stimulation of insulin release in type 2 diabetic GK rats and in non-diabetic W rats. The mechanism behind the *LM* effect is depended on L-type calcium channels, PKC and PKA systems, and exocytosis via G protein-coupled exocytosis and partially mediated by K-ATP channels of the β-cells. Therefore, *LM* is a promising nutraceutical product to restore glycemic homeostasis in the context of T2DM.

## Figures and Tables

**Table 1:** Effect of *LM* long-term treatment on the Oral Glucose Tolerance test performed at 0, 10 and 20 days of treatment in GK and W rats. Values of blood glucose (mM) determined at 0, 30, 60, 90 and 120 min and the area under the curve of glucose (mM/120 min), in each time point, are presented.

Time (min)	0	30	60	90	120	AUC
	Group 1. GK + *LM* 1000 mg/kg b.w.					
Day 0	7.8 ± 0.2	20.5 ± 0.2	19.0 ± 0.3	16.4 ± 0.2 ^#^	12.8 ± 0.4	1049.7 ± 26.9
Day 10	6.8 ± 0.2	19.4 ± 1.1	17.5 ± 0.4	14,4 ± 0.5 ^####^	11.9 ± 0.4	1004.8 ± 48.0
Day 20	6.4 ± 0.3	18.1 ± 0.2 ^##^	15.6 ± 0.1 ^####^	11.9 ± 0.4 ^####^	8.7 ± 0.4 ^####^	827.3 ± 24.8 ^##^
	Group 2. GK + Placebo					
Day 0	7.0 ± 0.2	21.3 ± 0.5	19.2 ± 0.3	17.5 ± 0.2	13.4± 0.4	1209.0 ± 39.0
Day 10	7.1 ± 0.6	20.4 ± 0.3	19.4 ± 0.3	17.6 ± 0.4	12.5 ± 0.4	1162.0 ± 70.0
Day 20	6.9 ± 0.2	19.9 ± 0.3	18.7 ± 0.2	16.7 ± 0.2	11.8 ± 0.7	1110.6 ± 19.3
	Group 3. W + *LM* 1000 mg/kg b.w.					
Day 0	4.1 ± 0.3	11.7 ± 0.4	9.8 ± 0.4	7.3 ± 0.3	6.1 ± 0.4	525.5 ± 35.2
Day 10	4.1 ± 0.1	10.5 ± 0.7 ^###^	9.1 ± 0.6 ^#^	6.9 ± 0.2	5.8 ± 0.5	453.5 ± 42.0
Day 20	4.1 ± 0.1	9.3 ± 0.1 ^####^	7.7 ± 0.1 ^####^	6.4 ± 0.1 ^####^	5.4 ± 0.1 ^###^	354.3 ± 12.2 ^#^
	Group 4. W + Placebo					
Day 0	3.7 ± 0.2	12.3 ± 0.1	10.1 ± 0.1	7.4 ± 0.1	6.1 ± 0.2	593.2 ± 15.0
Day 10	4.3 ± 0.3	12.1 ± 0.1	10.3 ± 0.1	7.5 ± 0.1	6.0 ± 0.2	534.5 ± 30.4
Day 20	4.6 ± 0.3	12.1 ± 0.3	10.2 ± 0.2	7.8 ± 0.2	6.3 ± 0.1	507.5 ± 40.1

Data are presented as means ± SEM (*n* = 6). ^#^ *p* < 0.05, ^##^ *p* < 0.01, ^###^ *p* < 0.001, ^####^ *p* < 0.0001 compared to placebo group at the same time point.

## Supplementary Materials

The following are available online at http://www.mdpi.com/2072-6643/10/7/933/s1, Figure S1, *LM* effect on insulin secretion was comparable with the effect of glibenclamide, Figure S2, Cytotoxic effect of *LM* on W pancreatic islets, Figure S3, Effect of *LM* long-term treatment on body weight, Figure S4, Effect on body weight of Wistar rats after 28 days of *LM* treatment, Figure S5. Effect on body weight of Wistar rats after 28 days of LM treatment, Table S1, Effect on hematological and biochemical parameters of Wistar rats after 28 days of *LM* treatment.
