# Supplementary material for: Lupinus mutabilis Extract Exerts an Anti-Diabetic Effect by Improving Insulin Release in Type 2 Diabetic Goto-Kakizaki Rats
Source: Nutrients. 2018 Jul 20;10(7):933. doi: 10.3390/nu10070933 (PMC6073986; doi:10.3390/nu10070933)
Supplement: Supplementary file 1 [file nutrients-10-00933-s001.pdf]

Supplementary Materials

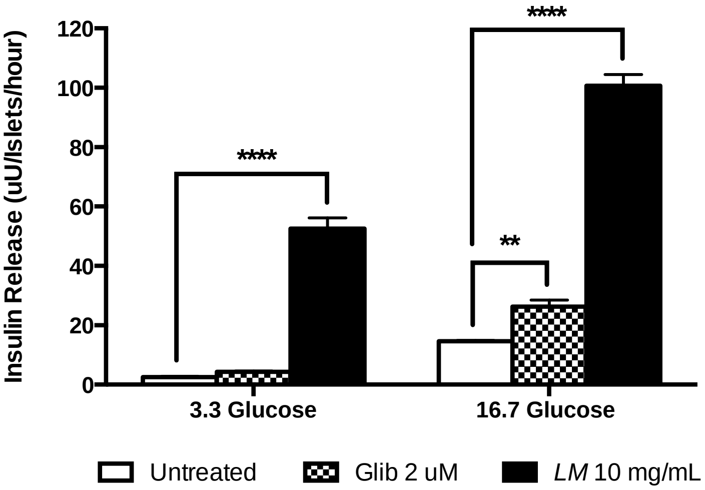

Figure S1. *LM* effect on insulin secretion was comparable with the effect of glibenclamide. Insulin secretion in GK islets was evaluated in low (3.3 mM) and high (16.7 mM) glucose in presence of a positive control Glibenclamide (2 µM) and *LM* (10 mg/mL). Insulin concentration was measured by RIA. Data are presented as means ± SEM (n = 3), of triplicates from three independent experiments. * $p < 0.05$, ** $p < 0.01$, *** $p < 0.001$, **** $p < 0.0001$ when compared to untreated islets.

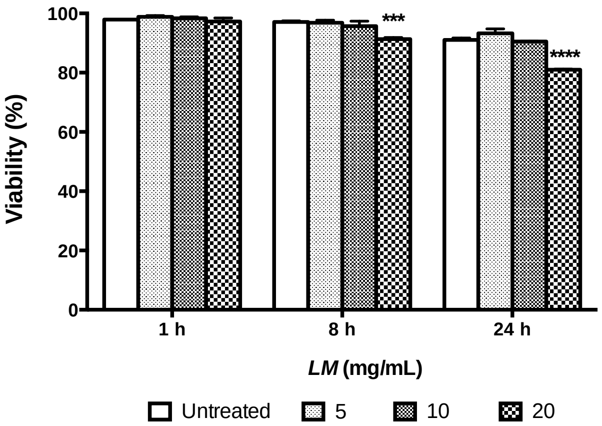

Figure S2. Cytotoxic effect of *LM* on W pancreatic islets. Panceatic islets of W rats were incubated in presence of *LM* extract (5, 10 and 20 mg/mL). Cell viability was measured by MTT assay after 1, 8 and 24h of treatment. Data are presented as means ± SEM (*n* = 3), of triplicates from three independent experiments. **$p < 0.05$, ** $p < 0.01$, *** $p < 0.001$, **** $p < 0.0001$* when compared to untreated islets.

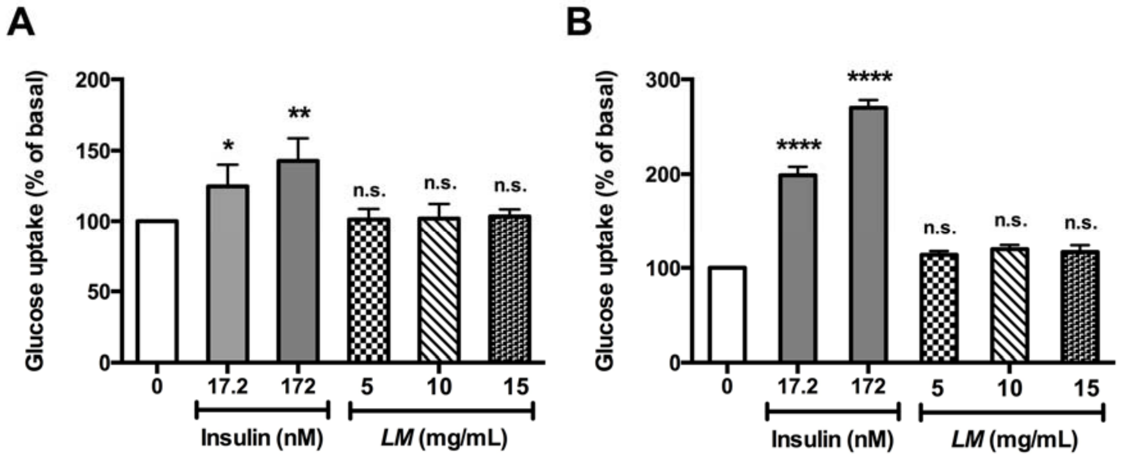

Figure S3. *LM* does not have effect on the glucose uptake in primary adipocytes. Glucose uptake in primary adipocytes was measured in disentegration per min/h (DPM/h) and the percentage of glucose uptake, relative to basal value was calculated. GK (A) and W rats (B) adipocytes were cultured in presence of *LM* (5-15 mg/mL) and insulin (17.2 and 172 nM) was used as positive control. Data are presented as means ± standard error of the mean (SEM) (n = 3), of triplicates from three independent experiments. **$p< 0.05$, ** $p<0.01$, **** $p<0.0001$* when compared to basal value.

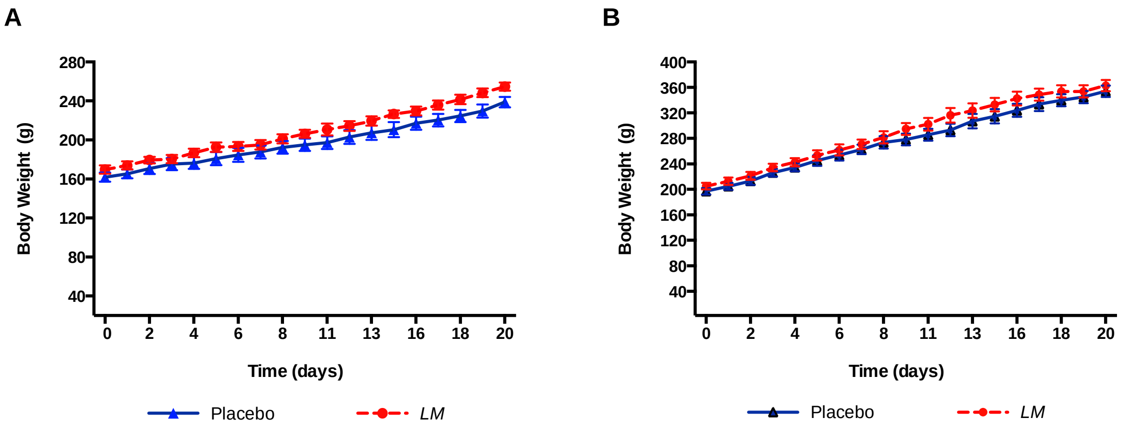

Figure S4. Effect of *LM* long-term treatment on body weight. The body weight was measured every day of *LM* treatment in GK (A) and W rats (B). Data are presented as means ± SEM (n = 6).

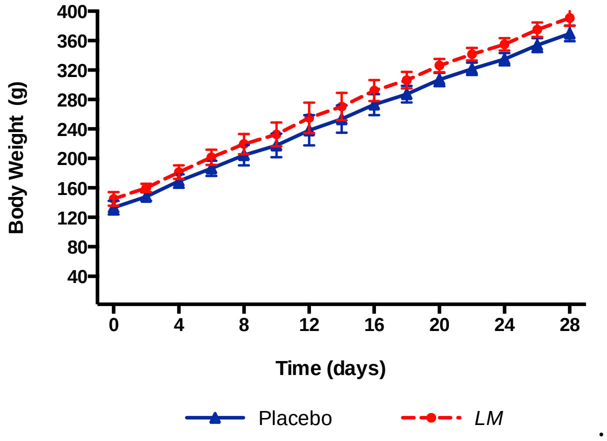

Figure S5. Effect on body weight of Wistar rats after 28 days of *LM* treatment. The body weight was measured every second day of treatment in W rats. Data are presented as means ± SEM (n = 6).

Table S1. Effect on hematological and biochemical parameters of Wistar rats after 28 days of *LM* treatment

Parameters	Placebo	*LM*
**Hematological**		
Hematocrit (%)	59.8 ± 1.7	59.0 ± 0.9
Hemoglobin (g/dL)	18.1 ± 0.5	17.0 ± 0.3
Red Blood Cells (x 10^6/μL)	9.0 ± 0.5	8.8 ± 0.5
White Blood Cells (x 10^3/μL)	11.4 ± 1.4	13.2 ± 1.1
Neutrophils (%)	36.0 ± 1.6	36.3 ± 2.5
Lymphocytes (%)	64.5 ± 3.0	53.7 ± 3.8
Monocytes (%)	2.0 ± 0.3	1.5 ± 0.2
Eosinophils (%)	1.7 ± 0.2	1.2 ± 0.2
Basophils (%)	0.0 ± 0.0	0.0 ± 0.0
**Biochemical**		
Triglycerides (mg/dL)	30.0 ± 4.3	29.1 ± 5.1
Cholesterol (mg/dL)	43.3 ± 3.0	51.2 ± 2.9
Glucose (μM)	6.8 ± 0.7	6.2 ± 0.7
Creatinine (mg/dL)	0.4 ± 0.02	0.3 ± 0.02
Alkaline Phosphatase (U/L)	118.0 ± 12.6	127.3 ± 10.8
Aspartate Aminotransferase (U/L)	126.0 ± 14.6	121.6 ± 15.4
Alanine Aminotransferase (U/L)	37.0 ± 3.1	39.4 ± 2.7

Data are presented as means ± SEM (*n* = 6).
